# Selenium-biofortified spent yeast cultivated in corn hydrolysate: antioxidant response and biomass production under aerobic and anaerobic conditions

**DOI:** 10.1007/s10123-025-00722-y

**Published:** 2025-10-14

**Authors:** Layna Amorim Mota, Rubens Perez Calegari, Alana Uchôa Pinto, Pietro Sica, Deoclecio Jardim Amorim, Ricardo Antunes Azevedo, Salete Aparecida Gaziola, Rafael Soares Douradinho, Antonio Sampaio Baptista, Valter Arthur

**Affiliations:** 1 Center for Nuclear Energy in Agriculture, University of São Paulo (CENA-USP), CEP 13416-000 Piracicaba, Brazil; 2 Department of Biological and Chemical Engineering , Aarhus University (BCE-AU) Gustav Wieds Vej 10D, Aarhus, Denmark; 3 Luiz de Queiroz College of Agriculture, University of São Paulo, Piracicaba, Brazil

**Keywords:** Glutathione system, Industrial fermentation, Lipid peroxidation, Oxidative stress, Reactive oxygen species, Selenized yeast

## Abstract

Spent yeast represents a promising opportunity for value-added applications. This study proposes its biofortification as a source of organic selenium (Se)-enriched supplements. Se is an essential component of the glutathione (GSH) system, playing a critical role in decomposing lipid peroxidation products and protecting cellular membranes. We evaluated the effects of sodium selenite (Na₂SeO₃) supplementation on enzymatic activity, oxidative stress markers, and biomass production of *Saccharomyces cerevisiae* Thermosacc^®^, cultivated in corn hydrolysate—a non-synthetic medium that provides a more realistic representation of industrial environments—under aerobic and anaerobic conditions. Antioxidant responses were assessed via glutathione peroxidase (GPx), glutathione reductase (GR), and glutathione S-transferase (GST) activities, while oxidative stress was measured through hydrogen peroxide (H₂O₂) and malondialdehyde (MDA) levels. Yeasts were grown with 0, 200, and 400 mg L^−1^ Na₂SeO₃. The highest enzymatic activities were observed in AE400 (GPx: 5.35 μmol mg^−1^, GR: 3.39 μmol mg^−1^, GST: 0.035 μmol mg^−1^), indicating enhanced antioxidant defenses under aerobic Se supplementation. However, increased Se concentrations also elevated H₂O₂ and MDA levels—especially in aerobic conditions—likely due to intensified ROS generation. Consequently, biomass production and growth parameters declined, suggesting an energy trade-off in which antioxidant defense is prioritized over cell proliferation. These findings highlight Se’s dual role as both an antioxidant and a pro-oxidant at elevated concentrations. This study advances understanding of yeast redox biology and supports the integration of Se-enriched yeast production into industrial fermentation as a sustainable strategy for generating high-value functional ingredients for food and feed applications.

## Introduction

Rising healthcare costs are leading many individuals to seek alternative ways to maintain their health and reduce medical expenses (Adadi et al. 2019). Thus, ensuring sufficient nutrient intake is one effective strategy, as it supports essential physiological functions (Rayman 2012). Contradictorily, the World Health Organization (WHO) identifies micronutrient deficiencies as a major global health concern, contributing to approximately 1.7 million deaths annually (Narwal et al. 2017).

Selenium (Se) is an essential micronutrient for both human and animal health, playing a vital role in metabolism, homeostasis, and immune function (Zhou et al. 2022). Se deficiency is associated with impaired immune responses, including reduced chemotaxis, phagocytosis, and respiratory burst activity. In macrophages, Se mitigates ROS-induced toxicity, limits pathogen replication, and enhances phagocytic function (Pecoraro et al. 2022), and several studies have demonstrated its anticancer properties (Davis et al. 2012).

Low Se concentrations in soils lead to inadequate Se accumulation in crops, making it difficult to meet dietary requirements without external supplementation (Mutonhodza et al. 2022). This deficiency contributes to widespread Se inadequacy, affecting an estimated one billion people globally (Zhang et al. 2019). Moreover, as Se is a non-renewable resource, its global scarcity—and associated health risks—is expected to intensify in the coming years (Qin et al. 2023). For these reasons, research on the biofortification of food and animal feed with Se has intensified in recent decades, aiming to enhance public health and food safety worldwide (Ogra et al. 2018; Mota et al. 2022; Kieliszek et al. 2022; Hachemi et al. 2023).

An alternative that merits further research is the use of yeast biomass to produce Se-enriched supplements, such as probiotics, particularly in organic forms, which are more bioavailable than their inorganic counterparts (Kieliszek et al. 2019). Fermentative processes generate large volumes of spent yeast, yet only a small portion is currently utilized for animal feed and an even smaller fraction for human consumption (Puligundla et al. 2020; Xavier et al. 2025). Consequently, increasing industrial interest in improving productivity, diversifying substrate use, and developing novel compounds has driven efforts to unlock the untapped potential of existing industrial yeast strains—particularly through their improvement into high-added-value biofortified probiotics (Da Silva Fernandes et al. 2022).

Yeasts are capable of converting inorganic selenium (Se) into bioactive organic forms that enhance cellular antioxidant defense mechanisms. These Se-enriched compounds have high added value and can be used in dietary supplements, pharmaceuticals, and functional foods to help combat oxidative stress. However, excessive intracellular Se accumulation can be toxic to most microorganisms (Lazard 2021), acting as a pro-oxidant with significant cytotoxic effects (Lee and Jeong 2012). Nevertheless, yeast, as a unicellular organism with rapid reproduction and high adaptability to environmental changes, is well suited for adaptive evolutionary strategies (Walker and Walker 2018; Sica et al. 2024b). Therefore, gradual increases in Se concentrations during cultivation could be employed to stimulate resistance mechanisms and enhance tolerance to Se toxicity.

Bioactive selenium (Se) contributes to the synthesis of selenoproteins, encoded by 25 eukaryotic genes (Hariharan and Dharmaraj 2020). Nearly all selenoproteins function as redox enzymes within or outside cells, each exhibiting distinct substrate specificities (Burk and Hill 2015; Ogra et al. 2018). These enzymes are essential for various physiological processes, including DNA synthesis, removal of toxic or signaling peroxides, reduction of oxidized proteins and membranes, redox signaling, thyroid hormone metabolism, and Se transport and storage. Among them, glutathione peroxidase (GPx) is one of the most critical, responsible for neutralizing hydrogen peroxide (H₂O₂) and organic hydroperoxides in both intracellular and extracellular environments (Mehdi et al. 2013; Arshad et al. 2021). Another beneficial effect of Se is its ability to increase the activity of glutathione S-transferase (GST) (Christensen et al. 1994), an enzyme that plays a key role in detoxification processes. In active yeast, GST can effectively mediate the excretion and detoxification of aflatoxins (Paul et al. 2015), as also demonstrated in in vivo studies with rats (Sica et al. 2024a). These findings highlight the critical role of selenium in cellular defense and detoxification, reinforcing its relevance as a target for biofortification strategies aimed at improving health and nutritional quality (González-Salitre et al. 2023).

Another important factor that may influence the process of Se biofortification in yeast is the significant metabolic difference between aerobic and anaerobic conditions. Aerobic metabolism generates up to 38 ATP molecules per glucose molecule, while anaerobic fermentation yields only 2 (Lagunas 1979). Under anaerobic conditions, yeast metabolism is directed primarily toward ethanol production, whereas aerobic conditions favor biomass generation and energy production. Although aerobic growth supports higher cell proliferation, it also leads to increased formation of reactive oxygen species (ROS) due to oxygen-dependent respiration (Ohmori et al. 1999). Despite these well-known metabolic distinctions, limited knowledge exists regarding the formation of selenoenzymes and the oxidative stress responses of Se-enriched yeasts adapted to high Se concentrations under different metabolic conditions.

Based on this background, we formulated the following hypotheses:

1. Increasing concentrations of sodium selenite (Na₂SeO₃) in corn hydrolysate will stimulate the activity of antioxidant enzymes (GPx, GR, and GST) in *Saccharomyces cerevisiae* Thermosacc^®^ strain, with higher enzyme activity expected under aerobic conditions due to enhanced energy availability and oxygen-dependent redox regulation.
2. Higher concentrations of Na₂SeO₃ will induce oxidative stress in yeast cells, leading to a significant reduction in cell viability and biomass production.

For this study, the *Saccharomyces cerevisiae* Thermosacc^®^ strain—commonly used in ethanol production—was enriched with Na₂SeO₃ and cultivated under both aerobic and anaerobic conditions using corn hydrolysate as the substrate. The objective was to investigate the effects of these metabolic conditions on biomass production and the glutathione-based antioxidant system, by assessing the activities of GPx, GST, and GR enzymes. Oxidative stress levels were evaluated by quantifying malondialdehyde (MDA) and hydrogen peroxide (H₂O₂) as lipid peroxidation markers.

Unlike previous studies that use synthetic media (Izawa et al. 1995; Manfredini et al. 2004; Kaur and Bansal 2006; Talbi et al. 2019), this study uses corn hydrolysate, better reflecting industrial conditions. Unlike synthetic media, which contain defined concentrations of glucose and salts, corn hydrolysate provides a complex nutrient profile, including sources of carbon and nitrogen, cofactors, fermentable sugars (glucose, maltose, and oligosaccharides), as well as amino acids, minerals, and vitamins (Wahjudi et al. 2023; Chang et al. 2025). Derived from the enzymatic or chemical hydrolysis of corn starch, this substrate is widely available and cost-effective, as maize is currently one of the most produced grains in the world (Erenstein et al. 2022).

Additionally, the adaptive response of yeast to elevated Na₂SeO₃ concentrations was examined under both metabolic regimes, offering insights into enzymatic performance and oxidative stress in a realistic fermentation scenario. This approach not only advances the understanding of the impact of selenium on antioxidant enzyme activity under industrially relevant conditions but also contributes to the development of functional yeast-based ingredients. Se-enriched yeasts are known to accumulate bioactive compounds such as selenomethionine, selenocysteine, and selenium-containing proteins, while simultaneously enhancing the activity of antioxidant enzymes (Kieliszek et al. 2019; González-Salitre et al. 2023; Hachemi et al. 2023; Zhang et al. 2025), (Zhang et al. 2025; Kieliszek et al. 2019; González-Salitre et al. 2023; Hachemi et al. 2023), thereby reinforcing their applications in nutrition and health.

## Materials and methods

### Corn hydrolysate

The corn hydrolysate was prepared in a continuously stirred tank reactor equipped with constant agitation and temperature control. Corn kernels were sourced from local suppliers in Piracicaba, Brazil, and ground into granules with a particle size of ≤ 2 mm. Prior to hydrolysis, the appropriate mass of ground corn and ultrapure water (MilliQ^®^) was measured and mixed at a ratio of 65 g of corn per 100 mL of water (Sica et al. 2021). The pH of the mixture was then adjusted to 5.8 using 5 M NaOH.

The suspension was then heated to 87 °C, and α-amylase was added at a concentration of 0.1% (w/w) in a water bath with temperature control, while the mixture was continuously agitated at 180 rpm for 150 min to induce starch gelatinization and dextrinization. Subsequently, the temperature was reduced to 65 °C, and amyloglucosidase was added at a concentration of 0.1% (w/w). The mixture was maintained at 65 °C under constant agitation (180 rpm) for an additional 150 min to complete the saccharification process, resulting in the final corn hydrolysate (Douradinho et al. 2023).

Following enzymatic hydrolysis, the hydrolysate was supplemented with macronutrients (10 g L^−1^ (NH₄)₂SO₄, 3 g L^−1^ KH₂PO₄, and 0.5 g L^−1^ MgSO₄) and micronutrients (4.5 mg L^−1^ ZnSO₄·7H₂O, 0.3 mg L^−1^ CoCl₂·6H₂O, 1 mg L^−1^ MnCl₂·4H₂O, 0.1 mg L^−1^ CuSO₄·5H₂O, 4.5 mg L^−1^ CaCl₂·2H₂O, 3 mg L^−1^ FeSO₄·7H₂O, 0.4 mg L^−1^ Na₂MoO₄·2H₂O, 1 mg L−^1^ H₃BO₃, 0.1 mg L^−1^ KI, and 0.1 mg L^−1^ biotin).

### Yeast adaptation

The *Saccharomyces cerevisiae* Thermosacc^®^ strain, a commercially available dehydrated active yeast commonly used in corn ethanol production, was sourced locally and stored dry without rehydration until the start of experimental trials. For Se biofortification, the yeast was gradually adapted to sodium selenite (Na₂SeO₃, 98% purity; Synth^®^, Brazil) through a stepwise exposure protocol. This adaptation involved incremental additions of Na₂SeO₃ (5 mg L^−1^) to the corn hydrolysate medium, with an initial concentration of 240 mg L^−1^ for 32 cycles until 400 mg L^−1^, allowing the yeast to grow under progressively increasing concentrations of the compound, following the procedure described by Mota et al. (2022).

### Cell propagation assays

A two-stage yeast propagation system was implemented under aerobic conditions using fed-batch fermentation in laboratory-scale stirred glass reactors. The setup was designed to evaluate the effects of sodium selenite (Na₂SeO₃) supplementation on yeast growth and selenium biofortification, using corn hydrolysate as the primary carbon source. Key process parameters—including temperature, pH, aeration, and feeding rate—were carefully controlled to simulate industrially relevant fermentation conditions. The detailed experimental procedures are presented in the subsections below.

### Aerobic conditions

Yeast propagation under aerobic conditions was carried out according to the treatments described in Table 1. Each treatment was conducted in five replicates using corn hydrolysate as the substrate, supplemented with the respective Na₂SeO₃ concentrations specified for each condition.

**Table 1:** Description of treatments for yeast cultivation under aerobic and anaerobic conditions

Treatment	Condition	Na_2_SeO_3_ (mg L^−1^) added to the growth medium
AE0	Aerobic	0
AE200		200
AE400		400
AN0	Anaerobic	0
AN200		200
AN400		400

The experimental setup consisted of three laboratory-scale stirred glass reactors placed inside an incubator operating at 200 rpm, with the temperature maintained at 30 ± 2 °C. Each reactor was connected to a computer-controlled peristaltic pump (Atlas Scientific, New York, USA) to regulate the feeding rate. Reactors were continuously stirred at 200 rpm, and the temperature was maintained at 30 ± 2°C.

For the aerobic condition treatments (AE), aeration was provided using compressed air (~ 21% O₂) delivered through a 2-µm diffusion stone (Humlegardens, Ekolager, Sweden) at a flow rate of 1 v v⁻^1^ min⁻^1^, ensuring a 1:1 ratio between aeration gas volume and reactor volume. This airflow was controlled via a mass flow controller (Brooks, Hatfield, USA). The substrate volume-to-aeration rate ratio remained constant throughout the process.

The yeast propagation process was divided into two stages. In the first stage, a preparatory step was performed to allow the yeast to adapt to the stress conditions of extremely low sugar concentrations (50–100 mg L^−1^), facilitating a metabolic shift. This phase began with the addition of 7% (wet basis) of a previously adapted inoculum to 300 mL of corn hydrolysate, with an initial yeast concentration of 1.7 × 10⁷ cells mL^−1^. The medium contained 5 g L^−1^ of glucose and the corresponding concentration of Na₂SeO₃, as described in Table 1. This stage lasted 4 h, until total glucose depletion. Following this, the culture was transferred to a second 2-L continuously stirred tank reactor bioreactor for the second propagation stage.

In the second stage, the yeast culture was inoculated into 300 mL of corn hydrolysate containing 100 mg L^−1^ of glucose and adjusted to pH 5.8. Propagation was carried out in a fed-batch system with an initial feed rate of 0.208 mL min^−1^. The feed rate was calculated based on Eq. (1), as described by Lim and Shin (2013):

1 $$Fs (t)=\frac{\mu \left(t\right)[X\left(t\right)V\left(t\right)]}{{Y}_\frac{x}{s}\left(t\right)({S}_{f-}{S}_{m})}$$

where:

*Fs* = Feed rate of the substrate (mL h^−1^).

*µ* = Specific growth rate (0.155 h^−1^).

[*x* (*t*) *v* (*t*)] = Cellular biomass in the reactor (g L^−1^).

*Y*_*x*/*s*_ = Biomass yield coefficient (0.5 g cell/g sugar).

*S*_*f*_ = Feed substrate concentration (g L^−1^);

*S*_*m*_ = Residual substrate concentration where yield is maximal (g L^−1^).

The second stage lasted 12 h, resulting in a total cultivation duration of 20 h. Subsequently, all material was centrifuged at 3000 g for 20 min at 4 °C and stored in a freezer (−  80 °C) for later analyses.

### Anaerobic conditions

The treatments evaluated in this stage were conducted in five replicates, using corn hydrolysate as the substrate supplemented with the respective Na₂SeO₃ concentrations specified for each treatment. Cultivation was performed in fed-batch mode until the total working volume of the bioreactor reached 2 L. Initially, 200 mL of wort containing 150 g L^−1^ of glucose and adjusted to pH 5.8 was added to the reactor and inoculated with 8% (wet basis) of a previously adapted yeast inoculum. One hour after the start of fermentation, feeding commenced using a peristaltic pump, delivering additional wort (150 g L^−1^ glucose) at a flow rate of 1.66 mL min^−1^ for 6.02 h, totaling 600 mL of hydrolysate.

Fermentation was conducted in three laboratory-scale stirred glass reactors, as previously described. The temperature was maintained at 30 ±  2 °C throughout the process. At the end of fermentation, the entire volume was centrifuged at 3,000 × *g* for 20 min at 4 °C, and the biomass was stored at –80°C for subsequent analyses.

### Analyses

#### Cellular performance parameters

Cell viability was determined by methylene blue staining according to the method described by Pierce (1970). The amount of biomass generated during the process was quantified based on dry weight, and the number of cells per mL was determined by direct counting using a Neubauer chamber, both as described by Zago et al. (1996). The biomass yield (*Y*_*x/s*_) was calculated according to Eq. (2) (Aiba et al. 1968):

2 $${Y}_{{~}^{x}\!\left/ \!{~}_{s}\right.}=\frac{Xf-Xi}{Si-Sf}$$

where *Xf* and *Xi* represent the final and initial biomass concentrations, respectively, while *Si* and *Sf* correspond to the initial and final substrate concentrations.

The specific growth rate (*µ*) was calculated based on the logarithmic variation in cell concentration over time (Eq. 3) (Aiba et al. 1968):

3 $$\mu =\frac{ln Nf-ln Ni}{\Delta t}$$

where *Ni* and *Nf* represent the initial and final cell concentrations (cells mL^−1^), respectively, and Δ*t* corresponds to the time interval (in hours).

The ethanol content was determined by the distillation of 25 ml of sample followed by density measurement, as described by (Calegari et al. 2023).

#### Enzymatic analyses

Yeast biomass was disrupted using a laboratory mill (Retsch^®^ MM 400, Retsch, Haan, Germany) with tungsten beads at 4°C. Test buffers provided with the commercial kit (Sigma-Aldrich^®^) were also used to assist in cell disruption during milling. Following disruption, the extracts were centrifuged at 11,000 × g for 5 min at 4 °C, and the resulting supernatants were transferred to new tubes and stored at −80 °C, as described by Kieliszek et al. (2019). The activities of glutathione peroxidase (GPx), glutathione reductase (GR), and glutathione S-transferase (GST) were calculated based on the protein content of each sample and expressed as µmol mg^−1^ protein. Protein concentration was determined using the Bradford method (Bradford, 1976), with bovine serum albumin (Sigma-Aldrich, Brazil) as the standard. Absorbance for all enzymatic analyses was measured using a Genesys 10S UV-Vis spectrophotometer (Thermo Scientific, USA).

**GPx activity**

GPx activity was measured according to the method of Inoue et al. (1999), with modifications for spectrophotometric analysis. The reaction mixture was composed of the following reagents: 550 µL of 100 mM potassium phosphate buffer (PBS), pH 7.0, containing 3 mM EDTA (prepared by dissolving 10.71 g of K₂HPO₄ and 5.24 g of KH₂PO₄ in 1 L of distilled water); 100 µL of glutathione reductase (GR, 0.24 U mL^−1^), prepared by diluting 230 units in 2 mL of 100 mM PBS, pH 7.0; 100 µL of reduced glutathione (GSH, 10 mM), prepared by dissolving 30.7 mg of GSH in 10 mL of 100 mM PBS, pH 7.0; and 25 µL of 1 mM sodium azide, prepared by dissolving 0.6501 mg of sodium azide in 10 mL of 100 mM PBS, pH 7.0.

For analysis, 775 µL of the reaction mixture was transferred into cuvettes for both test and blank samples and incubated at 37 °C for 5 min. Next, 25 µL of the sample was added to the test cuvette, and the reaction was initiated by adding 100 µL of 1.5 mM NADPH (prepared by dissolving 2.5 mg in 2 mL of 100 mM PBS, pH 7.0) and 100 µL of 1 mM H₂O₂ (prepared by diluting 15 µL of H₂O₂ in 100 mL of distilled water). The reaction was monitored for 1 min, and absorbance was recorded at 340 nm. For the blank, 100 mM PBS (pH 7.0, with 3 mM EDTA) was used in place of the sample.

GPx activity was calculated using Eq. (4), based on the decrease in absorbance at 340 nm, applying an extinction coefficient of 6.22 mM⁻^1^ cm^−1^:

4 $$Enzyme\;activity\;(U\;)=\frac{\triangle Abs}{6,22mM}\times dilution\;factor$$

where ΔAbs = change in absorbance at 340 nm.

**GR activity**

GR activity was determined spectrophotometrically at 30 °C following the methodology described by Gomes-Junior et al. (2006), with slight modifications. The following reagents were prepared in 100 mM phosphate buffer (PBS), pH 7.5 (Buffer A): (1) 1 mM DTNB (5,5′-dithiobis(2-nitrobenzoic acid)), (2) 1 mM GSSG (oxidized glutathione), and (3) 0.1 mM NADPH.

For the blank cuvette, the reaction mixture consisted of 1 mL of buffer A, 500 µL of DTNB solution, 100 µL of NADPH solution, 100 µL of GSSG solution, and 50 µL of buffer A. For the sample cuvette, 50 µL of the sample replaced the 50 µL of buffer A. The reaction was initiated by the addition of the sample, and GR activity was estimated by monitoring the reduction of GSSG, indicated by changes in absorbance at 412 nm over 1 min. Enzyme activity was expressed in µmol mg⁻^1^ protein.

**GST activity**

GST activity was measured using the following stock solutions: (1) 0.1 M phosphate buffer (PBS), pH 6.5; (2) 20 mM reduced glutathione (GSH), prepared by dissolving 0.123 g of GSH in 20 mL of PBS; and (3) 0.04 M CDNB (1-chloro-2,4-dinitrobenzene), prepared by dissolving 0.162 g in absolute ethanol and incubating at 30 °C.

For the assay, the reaction mixture consisted of 900 µL of PBS, 50 µL of GSH solution, 25 µL of sample, and 25 µL of CDNB solution. The components were mixed and allowed to stand for 20 s before absorbance was measured at 340 nm using a spectrophotometer, as described by Habig and Jakoby (1981).

GST activity was calculated using Eq. (5):

5 $$GST\;Activity\;(U\;mg^{-1}\;protein)=\frac{\triangle ABS}{9,6mM\;x\;sample\;volume\;(mL)\;x\;Protein\;Concentration\;(mg\;mL^{-1})}$$

where 9.6 mM^−1^ cm^−1^ is the molar extinction coefficient for GST activity using CDNB. The results were expressed as GST units per mg of protein.

#### Oxidative stress marker analyses

##### H_2_O_2_

The determination of hydrogen peroxide (H₂O₂) content was carried out according to the methodology described by Alexieva et al. (2001). For this analysis, 0.2 g of yeast biomass was homogenized in an ice bath with 2 mL of 0.1% trichloroacetic acid (TCA). The homogenate was transferred to a microcentrifuge tube and centrifuged at 13,000 × g for 10 min at 4°C. Subsequently, 0.2 mL of the supernatant was mixed with 0.2 mL of PBS and 0.8 mL of 1 M KI.

The reaction mixture was incubated in the dark for 1 h, after which the absorbance was measured at 390 nm using a spectrophotometer. For the blank, 0.2 mL of 0.1% TCA was used in place of the sample supernatant. The H₂O₂ content was quantified using a standard curve generated from known H₂O₂ concentrations, considering that 1 µmol of H₂O₂ corresponds to 34 µg.

##### MDA

The evaluation of malondialdehyde (MDA) content was performed according to the method of Heath and Packer (1968). A 0.2-g sample of fresh yeast biomass was homogenized in 2 mL of 0.1% trichloroacetic acid (TCA) containing approximately 20% polyvinylpolypyrrolidone (PVPP). The homogenate was centrifuged at 13,000 × g for 10 min at 4°C. Subsequently, 1 mL of 0.1% TCA was added to 0.25 mL of the supernatant, and the mixture was heated at 95 °C for 30 min. The reaction mixture was then rapidly cooled on ice for 10 min and left to rest at room temperature in the dark for 15 min.

Absorbance was measured at 532 nm and 600 nm using a spectrophotometer. MDA concentration was calculated using the extinction coefficient *ε* = 155 mM^−1^ cm^−1^, according to Eq. 6:

6 $$\text{MDA content ( mM }{\text{mL}}^{-1}\text{)} \, \text{=} \, \frac{\text{ABS (532 - 600)}}{\in 155000}$$

Results were expressed as micromoles of MDA per gram of fresh biomass (µmol g^−1^).

### Statistical analysis

Cell propagation and fermentation experiments were conducted using a completely randomized block design, consisting of three treatments with five replicates each, totaling 15 experimental units. The results were subjected to analysis of variance (ANOVA) using the *F*-test (*p* ≤ 0.05), and treatment means were compared using Tukey’s test at a 5% significance level. Statistical analyses and graph generation were performed using R software (R Core Team 2024) and Origin 2022b (64-bit), SR1, Version 9.9.5.171 (Academic).

## Results

### Effects of Se and metabolic conditions on yeast growth and biomass yield

Cultivation under different metabolic conditions (aerobic and anaerobic) was carried out using the *Saccharomyces cerevisiae* Thermosacc^®^ strain in corn hydrolysate medium supplemented with high concentrations of sodium selenite (Na₂SeO₃) at 200 and 400 mg L^−1^. The cellular performance observed under each condition is presented in Table 2.

**Table 2:** Cell propagation performance parameters of*Saccharomyces cerevisiae*Thermosacc^®^under aerobic conditions, including process duration (h), final ethanol content (% v/v), biomass produced (g L^−1^), cell concentration (cells mL^−1^), specific growth rate (*μ*, h^−1^), and biomass yield (*Yₓ*_*/s*_, g g^−1^)

Treatment	Process duration (h)	Final ethanol content (% v v^−1^)	Biomass produced (g L^−1^)	Cell concentration (cells mL^−1^)	*μ* (h^−1^)	*Y*_*x/s*_ (g g^−1^)
AE0	12	0.20 ± 0.03a	41.94 ± 0.85a	1.46 × 10^9^ ± 0.16a	0.159 ± 0.002a	0.50 ± 0.01a
AE200	12	0.46 ± 0.03b	31.00 ± 0.59b	8.27 × 10^8^ ± 0.17b	0.138 ± 0.001b	0.39 ± 0.01b
AE400	12	1.04 ± 0.03c	23.05 ± 0.56c	6.24 × 10^8^ ± 0,14c	0.118 ± 0.002c	0.32 ± 0.01c
AN0	9	7.97 ± 0.11a	10.24 ± 0.05a	3.3 × 10^8^ ± 0.11a	0.078 ± 0.000a	0.38 ± 0.02a
AN200	10	7.68 ± 0.09b	9.66 ± 0.23b	2.70 × 10^8^ ± 0.16b	0.068 ± 0.001b	0.31 ± 0.01b
AN400	12	7.50 ± 0.06c	9.35 ± 0.30b	2.14 × 10^8^ ± 0.1c	0.055 ± 0.001c	0.28 ± 0.01c

Different lowercase letters indicate significant differences within AE or AN groups (*p* < 0.05). No comparisons were made between aerobic and anaerobic treatments

Under aerobic conditions, all treatments showed statistically significant differences (*p* ≤ 0.05), with the highest specific growth rate (*μ*) observed in the control treatment AE0 (0.159 ± 0.002 h^−1^). As the Na₂SeO₃ concentration increased, a progressive decline in μ was observed, accompanied by an extended generation time. The lowest *μ* was recorded in treatment AE400 (0.118 ± 0.002 h^−1^), representing a reduction of approximately 25% compared to AE0. A similar trend was observed under anaerobic conditions, where treatments also differed significantly and displayed an inverse relationship between μ and Na₂SeO₃ concentration. Treatment AN0 exhibited the highest *μ* (0.078 ± 0.000 h^−1^), while AN400 showed the lowest (0.055 ± 0.001 h^−1^), corresponding to a reduction of approximately 30% relative to the control. When comparing the two metabolic conditions, it is evident that *μ* values under aerobic conditions were, on average, twice as high as those observed under anaerobic conditions.

All treatments also showed statistically significant differences (*p* ≤ 0.05) in terms of biomass yield (*Yₓ*_*/s*_) under both aerobic and anaerobic conditions (Table 2). The highest yield was obtained in treatment AE0 (0.50 ± 0.01 g g^−1^), approximately 24% higher than the anaerobic control AN0 (0.38 ± 0.02 g g^−1^). Even at the highest Na₂SeO₃ concentration (400 mg L^−1^), the aerobic condition maintained superior yields: AE400 reached 0.32 ± 0.01 g g^−1^, while AN400 achieved 0.28 ± 0.01 g g^−1^. These results underscore the significant impact of both Na₂SeO₃ concentration and metabolic condition on yeast cellular performance parameters.

**Fig. 1:**
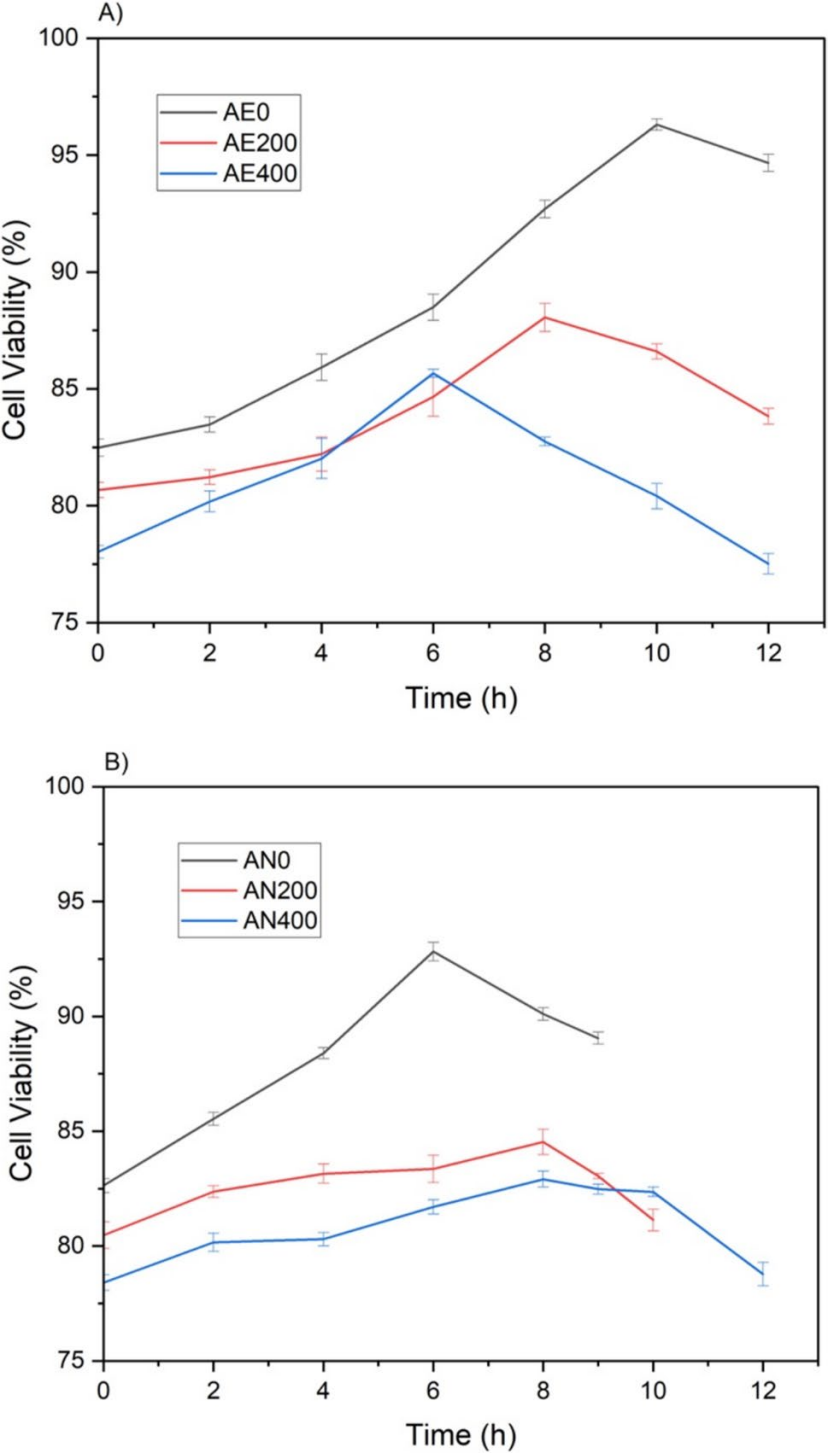
**A** Cell viability throughout the entire cultivation process of the treatments under aerobic conditions; **B** cell viability throughout the entire cultivation process of the treatments under aerobic conditions

Furthermore, as shown in Fig. 1A and B, which illustrate cell viability throughout the entire process for all treatments, a progressive decrease in viability was observed with increasing Na₂SeO₃ concentrations. Notably, however, the reduction in viability relative to the initial values did not exceed 5.4% in any treatment. This indicates that the prior adaptation of yeast cells to Na₂SeO₃ was effective in preserving cellular integrity throughout the cultivation process. Under aerobic conditions, final cell viability reached 94.66 ± 0.37% for AE0, 83.82 ± 0.34% for AE200, and 77.51 ± 0.44% for AE400. Under anaerobic conditions, viability values were 89.05 ± 0.26% for AN0, 81.13 ± 0.47% for AN200, and 78.78 ± 0.51% for AN400. As expected, the addition of sodium selenite negatively impacted cell viability, with control treatments (AE0 and AN0) exhibiting the highest values under both metabolic conditions. Notably, AE0 presented approximately 6% higher viability than AN0, highlighting the greater resilience of cells under aerobic conditions.

### Glutathione-related enzyme activity in biofortified yeast

**Fig. 2:**
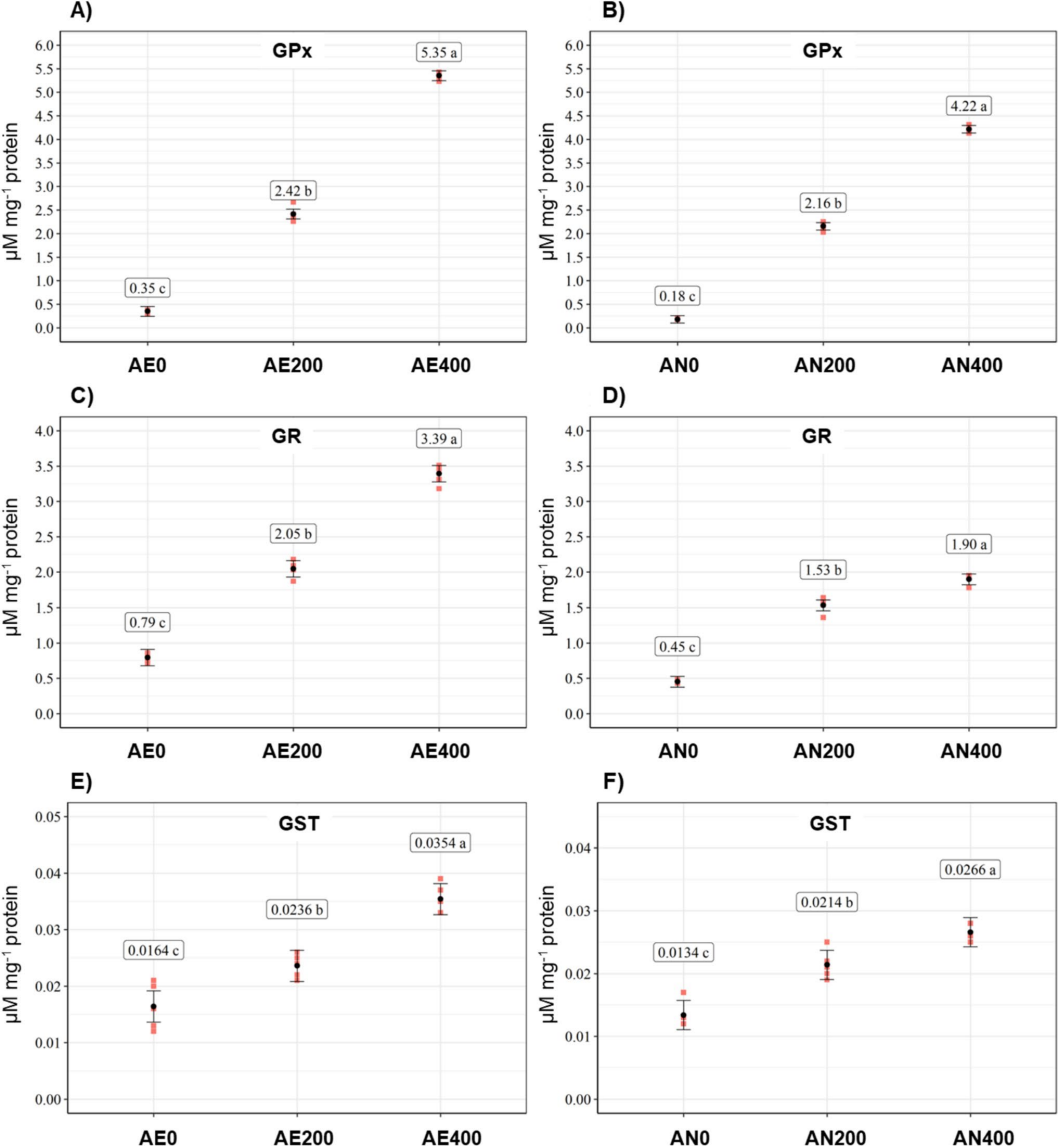
**A** quantification of GPx enzymatic activity in all treatments under aerobic conditions; **B** quantification of GPx enzymatic activity in all treatments under anaerobic conditions; **C** quantification of GR enzymatic activity in all treatments under aerobic conditions; **D** quantification of GR enzymatic activity in all treatments under anaerobic conditions; **E** quantification of GST enzymatic activity in all treatments under aerobic conditions; **F** quantification of GST enzymatic activity in all treatments under anaerobic conditions

Increasing the Na₂SeO₃ concentration in the medium, under both aerobic and anaerobic conditions, resulted in elevated activities of the enzymes GPx, GR, and GST, as shown in Fig. 2. These findings suggest that sodium selenite supplementation stimulated the antioxidant defense system, enhancing the activity of key enzymes involved in redox homeostasis.

All treatments under both aerobic and anaerobic conditions showed statistically significant differences in enzymatic activity (*p* ≤ 0.05). Under aerobic conditions, the highest enzyme activities were observed in treatment AE400, with values of 5.35 ± 0.08 µmol mg^−1^ for GPx, 3.39 ± 0.14 µmol mg^−1^ for GR, and 0.035 ± 0.003 µmol mg^−1^ for GST. Notably, GPx activity in AE400 was approximately 15 times higher than in the control treatment AE0 (0.35 ± 0.03 µmol mg^−1^), indicating a strong response to Na₂SeO₃ supplementation.

Similarly, under anaerobic conditions, yeast cells in treatment AN400 (400 mg L^−1^ Na₂SeO₃) exhibited elevated enzyme activity, with GPx, GR, and GST values of 4.22 ± 0.07, 1.90 ± 0.07, and 0.027 ± 0.001 µmol mg^−1^, respectively. As in aerobic conditions, GPx showed the highest activity among the enzymes measured.

When comparing the two metabolic conditions, enzymatic activities were consistently higher under aerobic conditions. For instance, in treatment AE400, GPx, GR, and GST activities were 26.77%, 78.42%, and 29.62% higher, respectively, compared to their counterparts in AN400. These results highlight the influence of oxygen availability on the antioxidant enzyme response in Se-enriched yeast.

### Oxidative stress markers

**Fig. 3:**
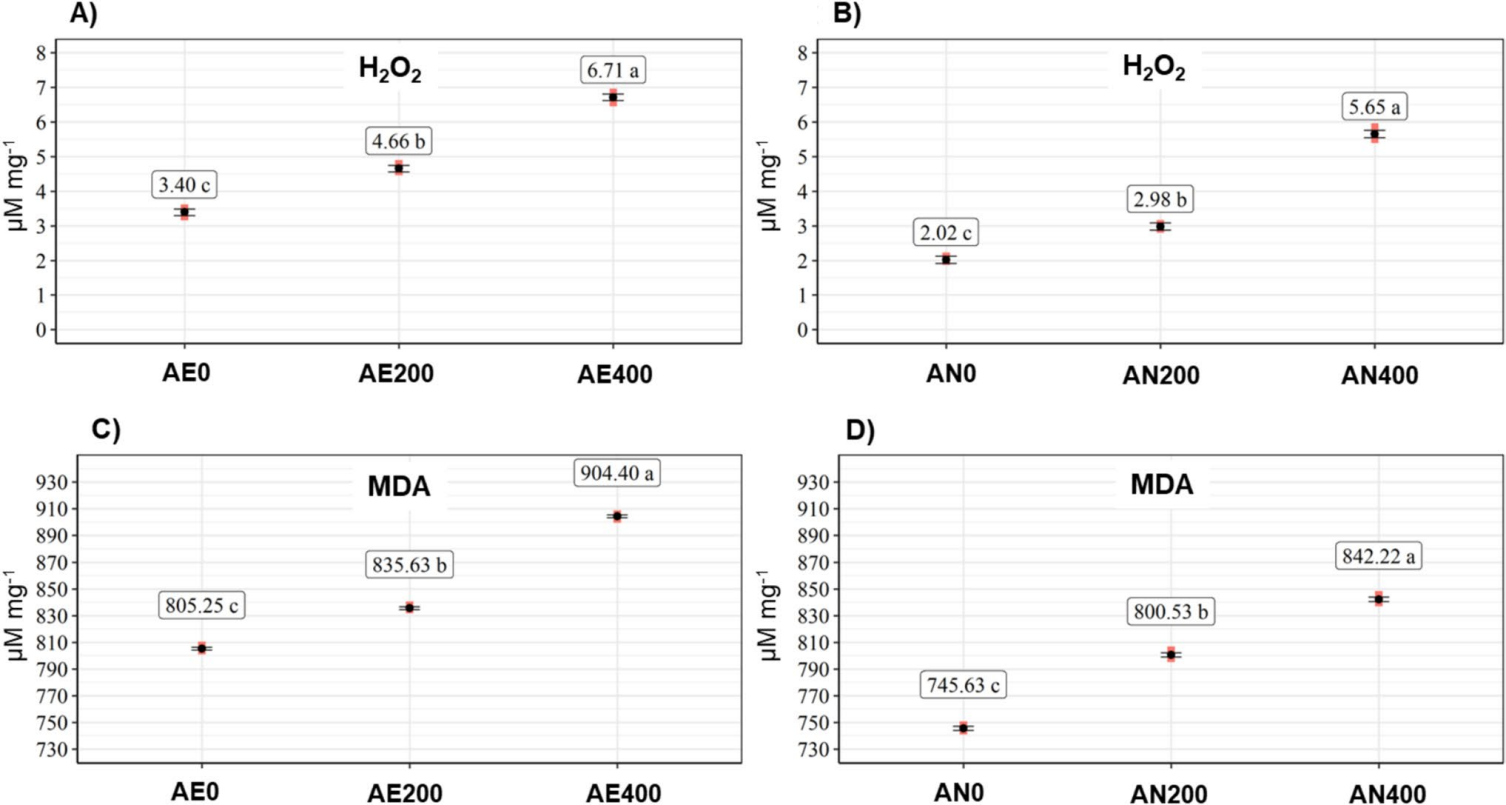
**A** Quantification of H₂O₂ in all treatments under aerobic conditions; **B** quantification of H₂O₂ in all treatments under anaerobic conditions; **C** quantification of MDA in all treatments under aerobic conditions; **D** quantification of MDA in all treatments under anaerobic conditions

The levels of oxidative stress markers—hydrogen peroxide (H₂O₂) and malondialdehyde (MDA)—in the culture media are presented in Fig. 3. Significant differences (*p* ≤ 0.05) were observed across treatments under both aerobic and anaerobic conditions.

In both metabolic conditions, increasing Na₂SeO₃ concentrations in the medium resulted in elevated levels of H₂O₂ and MDA. These findings suggest that, while Na₂SeO₃ stimulated antioxidant enzyme activity, it also induced oxidative stress—likely due to the toxic effects of high salt concentrations in the medium.

Under aerobic conditions, treatment AE400 showed the highest levels of oxidative stress markers, with H₂O₂ and MDA values reaching 6.71 ± 0.12 and 904.4 ± 1.79 µmol mg^−1^, respectively, representing increases of 97.35% and 12.31% compared to the control (AE0).

A similar trend was observed under anaerobic conditions, with the highest H₂O₂ and MDA levels also occurring in treatment AN400 (400 mg L^−1^ Na₂SeO₃). However, overall levels of oxidative stress markers were lower in anaerobic than in aerobic conditions. For example, H₂O₂ content in AN0 was 2.02 ± 0.06 µmol mg^−1^—40.58% lower than in AE0—while AN400 reached 5.65 ± 0.16 µmol mg^−1^, 15.79% lower than AE400.

## Discussion

### Selenium-induced antioxidant enzyme activation (H1)

The first hypothesis tested in this study was that increasing concentrations of Na₂SeO₃ in corn hydrolysate would stimulate the activity of antioxidant enzymes (GPx, GR, and GST) in *Saccharomyces cerevisiae* Thermosacc^®^ strain, with higher enzyme activity expected under aerobic conditions due to enhanced energy availability and oxygen-dependent redox regulation. The results found in this study confirm our first hypothesis.

One of the main motivations for promoting Se accumulation in yeast lies in the possibility of converting inorganic species—poorly absorbed by the human body—into bioactive organic forms with significantly higher bioavailability, given that inorganic Se comprises about 99% of dietary intake but has an absorption rate of only 10%. Se-enriched yeast represents a promising alternative for more effective dietary supplementation (González-Salitre et al. 2023). The predominant organic species are generally selenomethionine (SeMet), typically representing 60–85% of the total Se, followed by smaller amounts of selenocysteine (SeCys), depending on the cultivation conditions and yeast strain. Other organic compounds have also been reported, including methylselenocysteine (MeSeCys), seleno-methylselenocysteine (Se-MeSeCys), and seleno-adenosylselenohomocysteine (Se-AdoSeHCys), as well as selenium-containing proteins in which SeCys is incorporated as the 21 st amino acid (Rayman 2012; Kieliszek et al. 2015; Ogra et al. 2018; Hachemi et al. 2023). These bioactive forms, in addition to having higher bioavailability, are associated with beneficial health effects. Combined with the stimulation of antioxidant enzymes, these compositional features may confer significant functional value to the yeast biomass.

In this study, the highest levels of antioxidant enzyme activity were observed under aerobic conditions in treatment AE400, with values of 5.35 µmol mg^−1^ for GPx, 3.39 µmol mg^−1^ for GR, and 0.035 µmol mg^−1^ for GST. These findings suggest that oxygen availability plays a key role in amplifying Se-induced oxidative stress, thereby triggering an upregulation of antioxidant defenses as a compensatory response. During aerobic metabolism, the reduction of molecular oxygen leads to the formation of reactive oxygen species (ROS), including superoxide anion (O₂^−^), hydrogen peroxide (H₂O₂), and hydroxyl radicals (OH·), which can damage cellular components if not properly neutralized (Grant 2001).

Selenium plays a fundamental role as a structural component of selenoproteins and antioxidant enzymes such as glutathione peroxidase (GPx) and thioredoxin reductase (TrxR), which protect cells from oxidative damage caused by free radicals (Himeno and Imura 2000). Therefore, optimizing yeast growth and development conditions and understanding the biological effects of Se are essential to maximize the antioxidant potential of Se-enriched yeast (Kieliszek et al. 2019). In this study, we assessed the impact of Se supplementation by quantifying the activity of key antioxidant enzymes across different Na₂SeO₃ concentrations. A clear dose-dependent increase in the activity of GPx, glutathione reductase (GR), and glutathione S-transferase (GST) was observed, indicating that higher Se availability stimulates the yeast's antioxidant defense mechanisms.

Our findings are consistent with those reported by Kieliszek et al. (2019), who reported the impact of 40–60 mg L^−1^ Na_2_SeO_3_ on growth and the antioxidant system in *Candida utilis* ATCC 9950 and *S. cerevisiae* ATCC MYA-2200. The study found that higher Na_2_SeO_3_ concentrations in aqueous solutions resulted in increased activity of antioxidant enzymes, including GR, GPx, and GST, with peak levels of 0.78 µmol mg^−1^ protein for GR, 5.6 µmol mg^−1^ protein for GPx, and 0.072 µmol mg^−1^ protein for GST in *C. utilis* at 60 mg L^−1^ Na_2_SeO_3_.

In another study, Talbi et al. (2019) cultivated *S. cerevisiae* strain K310 in YPD medium supplemented with 0–5 mmol Na₂SeO₃ and observed that Se addition increased GPx activity, although superoxide dismutase (SOD) activity was not enhanced at higher Se concentrations. Similarly, Kaur and Bansal (2006) studied *S. cerevisiae* strain MTCC-1766 grown in YEPD medium with 19, 39, and 57 µM Na₂SeO₃. Their findings demonstrated a dose-dependent increase in GPx activity, from 16.15 ± 1.13 µmol mg^−1^ in the control to 106.63 ± 0.18 µmol mg^−1^ at the highest Se concentration. Increases in GR, GSH, and GST activities were also reported, underscoring Se's role in strengthening the yeast antioxidant defense system. Collectively, these studies support our findings by reinforcing the modulatory effect of Se on antioxidant enzyme activity in yeast.

The GSH system is ubiquitous across microorganisms, plants, and animals, and holds considerable biological significance due to its versatile physicochemical properties. It plays a central role in protecting cells against oxidative stress and xenobiotic toxicity (Fahey and Sundquist 1991; Couto et al. 2016). Casalone et al. (1988) observed that GPx activity is mediated by at least two distinct enzymes: a selenium-dependent GPx, which contains selenocysteine at its active site and is capable of reducing both H₂O₂ and organic hydroperoxides, and a selenium-independent GPx, which acts exclusively on organic hydroperoxides.

Glutathione S-transferases (GSTs) are a multifunctional enzyme family present across diverse organisms, playing a critical role in cellular detoxification. These enzymes primarily function by conjugating GSH with electrophilic compounds, thereby reducing their toxicity (Bai et al. 2004). A well-known example involves aflatoxin B1 (AFB1), which becomes mutagenic and carcinogenic following epoxidation to its reactive AFB1 8,9-epoxide form. This metabolite can form covalent bonds with nucleic acids or Schiff bases with cellular and microsomal proteins—such as guanine, methionine, and histidine—resulting in toxic and carcinogenic effects (Yiannikouris and Jouany 2002). GSTs produced by active yeasts can conjugate with the AFB1 epoxide, thereby preventing its interaction with target organs such as the liver and mitigating hepatic damage and its potential carcinogenic effects (Jeppesen et al. 2003; Paul et al. 2015). A recent study by Sica et al. (2024a) confirmed this protective mechanism through a radiolabeled experiment in Wistar rats, demonstrating the ability of active yeasts to mitigate aflatoxicosis via this detoxification pathway.

Unlike previous studies conducted with synthetic media, our research employed corn hydrolysate—a complex and industrially relevant substrate—offering a more realistic assessment of yeast performance under Se stress. To the best of our knowledge, this is the first study to investigate the adaptive responses and oxidative defense mechanisms of *Saccharomyces cerevisiae* exposed to high concentrations of Na₂SeO₃ in this medium. The observed upregulation of antioxidant enzymes and the yeast’s capacity to maintain cellular viability highlight its potential for biofortification under industrial conditions. These findings not only deepen our understanding of yeast stress physiology under high Na₂SeO₃ concentrations when cultivated in corn hydrolysate as a non-synthetic medium, but also open new avenues for integrating Se-enriched yeast as a high-value co-product in bioethanol production, with potential applications in animal feed, nutraceuticals, and functional foods aimed at addressing global Se deficiency.

### Selenium-induced oxidative stress and yeast growth inhibition (H2)

The second hypothesis tested in this study was that *higher concentrations of Na₂SeO₃* would induce oxidative stress in yeast cells, leading to a significant reduction in cell viability and biomass production. The results confirmed that all evaluated parameters were significantly affected by Na₂SeO₃ supplementation.

This study found statistical differences among treatments under both aerobic and anaerobic conditions—except for biomass production between treatments AN200 and AN400. Interestingly, although biomass levels did not differ significantly between these two anaerobic treatments, a marked extension in process duration was observed with increasing Na₂SeO₃ concentrations. Notably, fermentation in treatment AN400 extended to 12 h, suggesting that Se may influence fermentation kinetics. This observation implies that, despite the operational advantages of fed-batch systems for improving process efficiency (Li et al. 2011), elevated Se concentrations may hinder substrate uptake and CO₂ evolution, thereby delaying the completion of fermentation.

Elevated concentrations of Na₂SeO₃ may create a hostile environment for yeast cells, as previously reported by Hao et al. (2024). The osmotic pressure exerted by solutes in the medium is a critical factor that can impair both fermentative performance and biomass propagation. Under such osmotic stress, cellular metabolism may be hindered, reducing substrate conversion efficiency and leading to lower overall yields (Wang et al. 2013; Faramarzi et al. 2020). This interpretation is supported by the observed behavior of the specific growth rate (*μ*), with control treatments (AE0 and AN0) consistently showing the highest values within their respective metabolic conditions. Notably, during aerobic propagation, the carbon source is preferentially directed toward biomass formation (Martin and Chan 2024). In line with this, treatment AE0 yielded 0.50 ± 0.01 g of biomass per gram of sugar consumed, while AN0 reached 0.38 ± 0.02 g g^−1^, highlighting the greater efficiency of aerobic metabolism in supporting cell proliferation.

Taken together, these findings suggest that despite prior adaptive evolution to tolerate high Na₂SeO₃ levels, yeast performance was still negatively impacted as salt concentration increased—regardless of the metabolic condition. Nevertheless, aerobic cultivation consistently outperformed anaerobic fermentation, exhibiting both higher specific growth rates and biomass yields (*Y*_*x/s*_), further emphasizing its advantages for Se-enriched yeast production.

Our results suggest that, although selenium (Se) supplementation enhances antioxidant defenses, excessive Se concentrations can disrupt cellular redox homeostasis, ultimately triggering oxidative stress. This implies a metabolic trade-off for the yeast, as energy and resources are diverted from growth-related pathways to the synthesis of protective enzymatic compounds. While the adaptive process enabled yeast cells to survive in high Se environments, the resulting intracellular accumulation likely intensified osmotic stress. Moreover, Se can directly interfere with the cellular redox state by promoting the oxidation of thiol groups and indirectly increasing reactive oxygen species (ROS) formation. These changes diminish the cellular reducing environment, leading to oxidative damage. At the molecular level, Se reacts with thiol groups in proteins and with cysteine residues in reduced glutathione (GSH), forming intramolecular disulfide bonds, selenotrisulfide (S–Se–S), and selenosulfide (S–Se) linkages (Talbi et al. 2019). These modifications can disrupt cellular functions by altering the structure and activity of key receptors, enzymes, and transcription factors, further contributing to Se-induced cytotoxicity.

Reactive oxygen species (ROS) can cause extensive cellular damage, particularly when the cell's antioxidant defenses are insufficient to neutralize their effects. Sulfhydryl groups (−SH), especially those involved in the glutathione (GSH)/glutaredoxin and thioredoxin systems, play a central role in maintaining redox homeostasis during oxidative stress (Carmel-Harel and Storz 2000; Collinson et al. 2002).

Ohmori et al. (1999) demonstrated the influence of oxygen availability on oxidative stress responses in *Saccharomyces cerevisiae*, showing that aerobic conditions significantly enhanced GSH and cysteine levels. Their study reported increased activity of several key antioxidant enzymes, including γ-glutamylcysteine synthetase, glutathione synthetase, GST, GPx, GR, and γ-glutamyl transpeptidase. Notably, GST and GPx activities were higher under aerobic conditions (0.32 and 20.64 nmol mg^−1^ protein, respectively) compared to anaerobic conditions (0.26 and 17.27 nmol mg^−1^ protein). This enzymatic upregulation was mirrored by elevated H₂O₂ production, with aerobic conditions yielding 250 nmol H₂O₂ per 10 min per mg^−1^ protein, versus only 41.3 nmol under anaerobic conditions.

ROS are continuously generated in living organisms, including in mitochondrial membranes and through pathways such as microsomal electron transport (Barroso et al. 2011). These species can damage DNA, proteins, and lipids, posing a major risk factor for cellular aging and genetic instability. Among the various forms of oxidative damage, lipid peroxidation is recognized as a primary molecular mechanism by which ROS compromise membrane integrity and cellular function (Barroso et al. 2011; Lyu et al. 2017).

Lipid peroxidation is a key oxidative damage process initiated by free radicals in the hydrophobic regions of biological membranes. It proceeds in three distinct phases: initiation, propagation, and termination. In the initiation phase, highly reactive radicals—such as hydroxyl (•OH) or lipid hydroperoxide precursors (LOOH) fragmented by transition metals—generate lipid radicals (L•). During propagation, these lipid radicals react with molecular oxygen to form lipid peroxyl radicals (LOO•), which can abstract hydrogen atoms from DNA and proteins, ultimately forming new LOOH molecules. Termination occurs when two radicals combine to form stable, non-radical species. The breakdown of lipid hydroperoxides yields reactive aldehydes, such as malondialdehyde (MDA), and volatile hydrocarbons like ethane and ethylene, which are widely used as biomarkers for lipid peroxidation (Reginald Waldeck and Stocker 1996; Barroso et al. 2018).

Among ROS produced during aerobic metabolism, the superoxide anion (O₂•^−^) is the most prevalent. It undergoes dismutation to form hydrogen peroxide (H₂O₂), while also oxidizing Fe–S clusters in proteins. Picazo and Molin (2021) note that the most well-known ROS produced in aerobic metabolism is the superoxide anion radical (O_2_^−^), which is converted into H₂O₂ via dismutation. O_2_^−^ itself can oxidize Fe–S clusters, whereas H₂O₂ primarily reacts with the amino acids cysteine and methionine. On the other hand, the hydroxyl radical (•OH), which can be produced in reactions between H₂O₂ and metal ions (through the Fenton reaction), is considered the most powerful oxidant, as it can react with most biomolecules. However, its accumulation poses significant risks due to its role in initiating further radical reactions, such as the Haber–Weiss and Fenton reactions. Thus, tight regulation of H₂O₂ levels is crucial for maintaining redox balance.

According to Miyamoto et al. (2003), although oxidative stress is traditionally associated with cellular damage and genetic alterations, accumulating evidence suggests that reactive oxygen species (ROS) also function as intracellular messengers. They participate in gene regulation and signal transduction pathways, playing a key role in initiating adaptive responses to oxidative challenges. In the face of oxidative stress, organisms often temporarily halt energy-intensive processes—such as reproduction or macromolecule synthesis—to prioritize protective mechanisms. This adaptive shift involves the activation of key antioxidant systems, particularly enzymes like glutathione peroxidase (GPx). The cellular response to oxidative stress typically follows a structured sequence: ROS detection, activation of specific signaling cascades, and the transcriptional upregulation of genes encoding antioxidant enzymes. This process enhances the cell’s capacity to detoxify ROS, establishing a self-regulating negative feedback loop that restores redox homeostasis (Margis et al. 2008; Lushchak 2014).

Given the increased MDA levels observed in our study, lipid peroxidation emerges as a key marker of oxidative damage in Se-treated yeast cells. The elevated MDA concentrations confirm that high levels of Se induce oxidative stress that compromises membrane integrity. The combined effects of elevated Na₂SeO₃ concentrations and continuous O₂ exposure—both known to promote ROS formation—were evident in the quantitative analyses of H₂O₂ and MDA. Notably, the aerobic treatments showed significantly higher levels of both markers, indicating enhanced ROS generation under oxygen-rich conditions (Fig. 3).

When examining the metabolic conditions individually, the oxidative impact of Na₂SeO₃ was clear. In aerobic cultures, the AE400 treatment yielded the highest concentrations of H₂O₂ and MDA, reaching 6.71 μmol mg^−1^ and 904.4 μmol mg^−1^ of sample, respectively. Similarly, under anaerobic conditions, the AN400 treatment resulted in peak levels of 5.65 μmol mg^−1^ for H₂O₂ and 842.22 μmol mg^−1^ for MDA. These findings reinforce the conclusion that increased Se concentrations exacerbate oxidative stress, regardless of oxygen availability, although the effects are more pronounced under aerobic metabolism, in agreement with our first hypothesis.

According to Wonisch et al. (1998), oxidative stress–induced lipid peroxidation in biological systems results in the formation of a wide range of aldehydes due to the degradation of lipid hydroperoxides. Numerous studies have highlighted the critical role of GSH in protecting cells against oxidative damage. Izawa et al. (1995) investigated the involvement of intracellular GSH in *Saccharomyces cerevisiae* responses to hydrogen peroxide (H₂O₂). Yeast strains (S288C, YNN27, and YH-I) were exposed to 0.2 and 2 mM H₂O₂, with additional treatments using L-buthionine sulfoximine (BSO) and CDNB to inhibit GSH activity. The results showed that GSH depletion significantly increased cellular sensitivity to H₂O₂, confirming the protective role of intracellular GSH in oxidative stress response.

Similarly, Manfredini et al. (2004) studied *S. cerevisiae* mutants deficient in superoxide dismutase genes (sod1Δ, sod2Δ, and the double mutant) subjected to H₂O₂ stress (0.5–10 mM) during the stationary phase. In sod1Δ mutants, GSH levels increased by 200–260%, whereas GPx levels rose by 25–42% in sod2Δ and double mutants, depending on the H₂O₂ concentration. These findings suggest that yeast resistance to oxidative stress relies on GPx induction and that GSH functions as a primary antioxidant defense mechanism, particularly in sod1Δ mutants. Abegg et al. (2010) further reinforced the relevance of GSH-related enzymes by evaluating oxidative stress resistance across eight *Candida* species treated with 0.5 mM H₂O₂. Mild oxidative stress promoted adaptive responses in all strains through the induction of at least one of the antioxidant enzymes tested (GPx, SOD, and catalase). Notably, *C. famata* 62,894 showed the highest GPx activity, reaching 898.3 nmol mg^−1^ protein—an increase of 180% relative to the untreated control. Together, these studies underscore the central role of the GSH system and associated enzymes in microbial defense against oxidative damage.

Taken together, our results reinforce the critical role of the glutathione system—particularly GPx, GR, and GST—in mediating yeast responses to oxidative stress induced by Se exposure. The upregulation of these enzymes in Na₂SeO₃-supplemented cultures, especially under aerobic conditions, highlights the yeast’s adaptive capacity to maintain redox homeostasis despite elevated ROS levels and lipid peroxidation. These findings not only provide valuable insight into the cellular mechanisms underlying oxidative stress resistance in selenium-enriched yeast but also underscore the potential of adaptive cultivation strategies to enhance antioxidant capacity in biotechnological applications, including functional food development and value-added byproducts in ethanol production.

### Practical applications

To meet the demands of a growing global population (FAO 2009), it is essential to improve and integrate production systems within a circular and sustainable context, particularly in the energy and food sectors (Elobeid et al. 2013; Sanguino et al. 2020). This study proposes incorporating probiotic yeast production into industrial fermentation processes to generate *Saccharomyces cerevisiae* enriched with organic selenium—a form that is more bioavailable than its inorganic counterpart and associated with enhanced antioxidant enzyme activity—offering significant health benefits, including the potential to mitigate aflatoxicosis (Sica et al. 2024a).

Fermentation processes using *S. cerevisiae* are already well established in the food, beverage, and bioethanol industries (Crumplen et al. 1988), where spent yeast is typically treated as a low-value byproduct (Puligundla et al. 2020; Xavier et al. 2025). Our findings suggest that a portion of this biomass can be strategically redirected to diversify the product portfolio (Da Silva Fernandes et al. 2022) and be valorized as a high-added-value ingredient for the functional food and animal feed sectors.

In countries where ethanol production from sugarcane or corn is already established—such as Brazil and the USA (UNICAdata 2025)—this strategy could provide an opportunity to diversify industrial outputs. The USA currently leads global ethanol production with around 60 billion liters annually, while Brazil produced 36.8 billion liters in 2024, with corn-based ethanol now accounting for 20% of this volume since its initial implementation in 2017 (UNICAdata 2025). Additionally, sugarcane-producing countries such as India are expected to scale up their bioethanol capacity in the coming years (Dey et al. 2023).

Therefore, this study highlights the feasibility of integrating the production of Se-enriched probiotic yeast into existing food and alcohol beverage infrastructure, besides biofuel infrastructure, aligning industrial bioeconomy goals with public health and food security priorities.

## Conclusions

This study demonstrates that *Saccharomyces cerevisiae* Thermosacc^®^ can adapt to elevated concentrations of sodium selenite (Na₂SeO₃), leading to the biosynthesis of antioxidant enzymes and the intracellular accumulation of organic Se compounds. The results confirm that Se-induced oxidative stress triggers a robust cellular response—particularly under aerobic conditions—with significant increases in GPx, GR, and GST activities. However, Se supplementation also elevated levels of oxidative stress markers (H₂O₂ and MDA) and reduced growth-related parameters, indicating a metabolic trade-off in which antioxidant defenses are upregulated at the expense of biomass production. These findings contribute to a deeper understanding of yeast redox biology and the enzymatic mechanisms underlying microbial adaptation to micronutrient stress, providing a scientific basis for Se biofortification strategies.

Moreover, this study offers practical insights for industry stakeholders: producing Se-enriched yeast biomass using corn hydrolysate under controlled fermentation presents a sustainable and scalable pathway to generate high-value ingredients for functional foods and animal feed. This positions Se-enriched *S. cerevisiae* as a promising co-product in the bioethanol industry, adding nutritional and economic value to existing fermentation processes.

## Data Availability

No datasets were generated or analysed during the current study.
